# The Relationship between Neighborhood Immigrant Composition, Limited English Proficiency, and Late-Stage Colorectal Cancer Diagnosis in California

**DOI:** 10.1155/2015/460181

**Published:** 2015-10-04

**Authors:** Cynthia M. Mojica, Beth A. Glenn, Cindy Chang, Roshan Bastani

**Affiliations:** ^1^Department of Epidemiology and Biostatistics, Cancer Therapy & Research Center, School of Medicine, Institute for Health Promotion Research, The University of Texas Health Science Center at San Antonio, 7411 John Smith Drive, Suite 1000, San Antonio, TX 78229, USA; ^2^Center for Cancer Prevention and Control Research, Jonsson Comprehensive Cancer Center, Kaiser Permanente Center for Health Equity, Fielding School of Public Health, University of California, Los Angeles, 650 Charles Young Drive South, Room A2-125 CHS, Los Angeles, CA 90095-6900, USA; ^3^Center for Cancer Prevention and Control Research, Jonsson Comprehensive Cancer Center, University of California, Los Angeles, 650 Charles Young Drive South, Room A2-125 CHS, Los Angeles, CA 90095-6900, USA

## Abstract

Despite the availability of effective early detection technologies, more than half (61%) of colorectal cancers in the United States and 55% in California are identified at an advanced stage. Data on colorectal cancer patients (*N* = 35,030) diagnosed from 2005 to 2007 were obtained from the California Cancer Registry. Multivariate analyses found a relationship among neighborhood concentration of recent immigrants, neighborhood rates of limited English proficiency, and late-stage colorectal cancer diagnosis. Hispanics living in neighborhoods with a greater percentage of recent immigrants (compared to the lowest percentage) had greater odds (OR 1.57, 95% CI 1.22, 2.02) of late-stage diagnosis whereas Hispanics living in neighborhoods with the highest percentage of limited English proficiency (compared to the lowest percentage) had lower odds (OR .71, 95% CI .51, .99) of late-stage diagnosis. These relationships were not observed for other ethnic groups. Results highlight the complex relationship among race/ethnicity, neighborhood characteristics, and colorectal cancer stage at diagnosis.

## 1. Introduction

Colorectal cancer (CRC) is the second-leading cause of cancer death and the third-most common type of cancer among men and women in the United States [1]. In 2013, an estimated 142,820 new cases of colorectal cancer will occur, causing 50,830 deaths [1]. Similar to most other cancers, colorectal cancer survival and mortality are linked to stage of disease at diagnosis [2–5]. The 5-year survival rate drops from 90% for those diagnosed with early-stage colorectal cancer to 68% for regional spread (spread to adjacent organs and lymph nodes) and 10% for distant metastases [1]. Despite the availability of effective early detection technologies, more than half (61%) of colorectal cancers in the United States and 55% in California are identified at an advanced stage [1, 6]. Given the availability of effective colorectal cancer screening tests, communities across the USA are unnecessarily suffering from a disease for which early detection has proven effective. And as with many diseases, racial and ethnic minority groups share a disproportionate burden of late-stage colorectal cancer diagnoses. Numerous studies have found that African-Americans, Latinos, and various Asian subgroups are often diagnosed at later stages of diseases [1, 4, 7–11] and have lower survival and higher mortality rates compared to Whites [5, 9, 10, 12, 13].

Research exploring the reasons for diagnosis of late-stage colorectal cancer has implicated determinants at the individual and community level. Most studies to date have focused on individual characteristics such as low socioeconomic status [14–17] and lack of health insurance [1, 18]. Fewer studies have examined the relationship between community characteristics and late-stage colorectal cancer. Community factors associated with a greater likelihood of late-stage CRC diagnosis include living in rural [19] and medically underserved areas [20], great distances to cancer centers [21], and low neighborhood socioeconomic status, generally measured by income, education, composite measures of socioeconomic status, contextual poverty, and social deprivation [22–25]. However, prior studies have not examined how the proportion of recent immigrants within a community or the level of English proficiency among community residents influences stage at diagnosis for CRC. California provides an ideal setting in which to investigate these factors, given that 27% of residents are immigrants [26] and 6.9 million residents are considered having limited English proficiency, that is, having limited ability to read, write, speak, or understand English [27]. Research indicates that limited English proficiency results in difficulty accessing primary, preventive, and public health services [28–33], and limited English proficient individuals are more likely to receive low quality of care [34] and experience delays in care [35] once they access the system. Research on the effect of immigrant enclaves on health has produced conflicting results. Some studies suggest that immigrants tend to live in poor neighborhoods and that neighborhood poverty may have a detrimental effect on health including low birth weight and physical activity level [36, 37]. Other researchers found that a high concentration of immigrants in one area may shield individuals from the detrimental effects of poverty [38]. The prevailing thought is that ethnic/immigrant enclaves are protective of Latino health as enclaves provide opportunities to foster social relationships [39, 40]. A recent study found that Latinos living in neighborhoods with high concentrations of Latinos and immigrants were more socially integrated and had large, diverse networks [41]. However, no prior research has examined the effect of neighborhood concentration of recent immigrants or neighborhood level measures of limited English proficiency on CRC stage at diagnosis.

Therefore, the purpose of this study was to examine the relationship between neighborhood concentration of recent immigrants and neighborhood level rates of limited English proficiency in relation to late-stage CRC diagnosis in California after controlling for individual and other neighborhood-level factors.

## 2. Methods

### 2.1. Data Sources

Data on colorectal cancer cases diagnosed in California between 2005 and 2007 were obtained from the California Cancer Registry (CCR), a large, population-based cancer registry with information on all newly diagnosed cancer cases in California. The CCR is a collaboration of the California Department of Public Health, the Public Health Institute, the California Association of Regional Cancer Registries, the Centers for Disease Control National Program for Cancer Registries, and National Cancer Institute's Surveillance, Epidemiology, and End Results Program. CCR data were obtained geocoded to the census tract.

Data on neighborhoods (i.e., census tracts) were obtained from the RAND Data Core in the Center for Population Health and Health Disparities, National Institutes of Health Center for Population Health and Health Disparities. The RAND Data Core is a data resource center available to researchers and community-based organizations interested in how neighborhoods affect health, and houses all measures from the 2000 U.S. decennial census geocoded to the census tract.

### 2.2. Study Population

**Figure 1:**
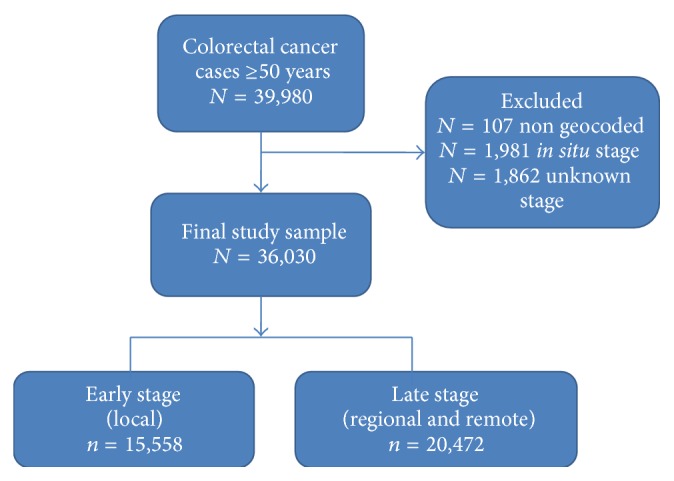
Colorectal cancer cases from California Cancer Registry (2005–2007) and final study sample.

Between 2005 and 2007 a total of 39,980 individuals aged 50+ were diagnosed with colorectal cancer in California (see Figure 1). From among these cases, 3,950 were excluded: 107 were not geocoded; 1,981 were diagnosed as* in situ*; and 1,862 were diagnosed as* unknown *stage. Thus, the final sample size was 36,030 cases across 6,617 census tracts (mean of 5.45 and median of five participants per census tract, range 1–67), with 93% of cases living in urban areas.

### 2.3. Variables

The outcome measure for this study was colorectal cancer stage at diagnosis, a derived variable created by the CCR. The derived variable is coded in accordance with guidelines from the CCR and the National Cancer Institute's Surveillance Epidemiology and End Results Program:* in situ*, localized, regional by direct extension, regional by lymph nodes, regional by direct extension and lymph nodes, regional (no lymph nodes), remote, and unknown. For this analysis, stage of colorectal cancer at diagnosis was coded as a binary variable (early versus late stage). Early-stage colorectal cancer was defined as cancer at a “localized” stage, whereas late-stage colorectal cancer was defined as cancer diagnosed at a “regional or remote” stage.

Factors examined as potential predictors of stage at diagnosis included both individual and neighborhood-level measures. Individual-level measures obtained from the California Cancer Registry for each colorectal cancer case were age at diagnosis (50–75, 75+), sex, race/ethnicity (White, Hispanic/Latino, Black, and Asian), marital status when patient was diagnosed (married versus unmarried), and health insurance (uninsured, Medicaid, Medicare, military/veterans, unknown, and private). Neighborhood measures obtained from the 2000 U.S. Census were percentage of recent immigrants (year of entry: 1995 to March 2000); percentage of limited English proficiency (speak English “well, not well, or not at all”); median household income; and percentage neighborhood deprivation. The neighborhood deprivation summary measure (index/scale) was created by calculating the average percentages of population ≥ 25 years of age without a high school diploma, population receiving public assistance, households with children headed by females, and male population aged 16 and over who are unemployed.

### 2.4. Statistical Analysis

Descriptive analyses were performed to summarize the characteristics of the study population. Quartiles of the neighborhood measures were used for all the analyses. Associations between stage at diagnosis and individual and neighborhood characteristics were examined using chi-square tests. Bivariate analyses were also used to examine the effects of each variable on stage at diagnosis. Multivariate analyses were used to examine the independent effects on stage at diagnosis. Generalized estimating equation logistic regression modeling was used to account for the potential correlation between participants residing in the same neighborhood. Analysis was performed using SAS (Windows version 9.1).

## 3. Results

### 3.1. Demographic Characteristics

Table 1 presents descriptive information on the 36,030 colorectal cancer cases diagnosed in California between 2005 and 2007. The sample consisted of 66% White, 15% Hispanic, 12% Asian, and 7% African-American cases. Most cases (62%) were aged 50–75 years, which is consistent with national data regarding age at diagnosis. Slightly over half were married and most had either private insurance (46%) or Medicare (45%). Only 6% were on Medicaid and 2% reported having no insurance.

### 3.2. Frequency of Late versus Early Stage at Diagnosis

Overall, the sample contained 20,472 (57%) cases of late-stage and 15,558 (43%) cases of early-stage colorectal cancer. In unadjusted analyses, the proportion of late-stage cancer differed significantly by all variables, with the exception of age.

### 3.3. Association of Stage at Diagnosis with Individual- and Neighborhood-Level Characteristics in Multivariate Analyses

Table 2 presents the relationship between stage at diagnosis and individual/neighborhood characteristics from multivariate regression analyses. The following independently predicted stage at diagnosis in adjusted analyses.

#### 3.3.1. Percentage Recent Immigrants

Hispanics who live in neighborhoods with a greater percentage of recent immigrants had greater odds of being diagnosed with late-stage colorectal cancer compared to Hispanics who lived in neighborhoods with the lowest percentage of recent immigrants.

#### 3.3.2. Percentage Limited English Proficiency

Hispanics who live in neighborhoods with the highest percentage of limited English proficiency had lower odds of being diagnosed with late-stage colorectal cancer compared to Hispanics who live in neighborhoods with the lowest percentage of limited English proficiency.

#### 3.3.3. Health Insurance

Non-Hispanic Whites and Asians with no insurance, Medicaid, or “unknown” insurance had higher odds of being diagnosed with late-stage colorectal cancer compared to those with private insurance. Hispanics with no insurance or Medicaid had higher odds of being diagnosed with late-stage colorectal cancer compared to those with private insurance, whereas Hispanics with military/veterans insurance had lower odds of being diagnosed with late-stage colorectal cancer. Only African-Americans with Medicaid had higher odds of being diagnosed with late-stage cancer compared to those with private insurance.

#### 3.3.4. Median Household Income

Income seemed to be predictive of stage at diagnosis for only Asians. Asians living in neighborhoods with a higher median household income had lower odds of being diagnosed with late-stage colorectal cancer compared to Asians living in the neighborhoods with the lowest median household income.

## 4. Discussion

This study merged California Cancer Registry and U.S. Census data to examine individual- and neighborhood-level predictors of late-stage colorectal cancer in California. Results showed late-stage colorectal cancer diagnosis was predicted by a greater proportion of recent immigrants living in a neighborhood, lower proportion of limited English proficiency in the neighborhood (for Hispanics), and median household income of the neighborhood (for Asians). Lack of health insurance at the individual-level, for all groups except African-Americans, also remained an independent predictor of late-stage colorectal cancer.

### 4.1. Neighborhood Percentage of Recent Immigrants

We found no studies assessing the relationship between percent recent immigrants in a neighborhood and late-stage colorectal cancer diagnosis. However, our results are similar to those in a study focused on breast cancer stage at diagnosis: authors found that an increased concentration of immigrant populations within neighborhoods contributed to risk of late-stage diagnosis of breast cancer [42]. Part of the explanation may be related to the nature of neighborhoods with large proportions of recent immigrants. These neighborhoods are generally transitory in nature and immigrants come and go as they arrive and acculturate to this country. Individuals living in such neighborhoods (with little to no social support or networks) are less likely to seek screening, which in part may contribute to later stage at diagnosis. Thus, recent immigrants do not benefit from the protective health effects of social capital.

### 4.2. Neighborhood Percentage of Limited English Proficiency

We expected to find higher late-stage colorectal cancer in neighborhoods with a higher percentage of limited English proficiency, yet we found that neighborhoods with a higher percentage of limited English proficiency had lower odds of late-stage disease. The literature on access to health care may help explain this finding. Recent research among U.S. Hispanics has found that neither English or Spanish language has an effect on determining access to care [43] or that Mexican-American immigrants have better access to care when living in areas with more Spanish speakers or more Hispanic immigrants [40]. A likely explanation may be that, living in areas where a majority of individuals speak the same language or where there are large groups of people with the same background, people form strong social networks [40, 44]. These bonds may increase the probability that health information is disseminated among the groups or neighborhood. A number of studies at the individual-level among Latina women have found that the presence of social networks is related to getting preventive care and cancer screenings [45, 46]. This may be the case in California: neighborhoods with a higher percentage of limited English proficiency may not necessarily be transitory in nature but have higher social cohesion and social capital that has been shown to be protective of health. Additionally, such neighborhoods may benefit from resources and the presence of local organizations and clinics that assist minorities and immigrants, including employing physicians who can communicate in Spanish [40]. Title VI of the 1964 Civil Rights Act requires entities receiving federal funds to provide language assistance to persons with limited English proficiency [47].

### 4.3. Health Insurance and Median Household Income

Our result on health insurance status shows that individuals with no insurance, Medicaid, and “unknown” insurance are more likely to be diagnosed with late-stage colorectal cancer compared to individuals with private insurance, which is similar to what has been found in other studies, namely, that uninsured and Medicaid patients are more likely to present with late-stage colorectal cancer [16, 18]. Also, we expected that individuals living in neighborhoods with higher incomes would have early stage at diagnosis, and we only saw this for Asians. While there is not much in the literature to help explain this finding, one study examining neighborhood income and Asian ethnicity with respect to liver cancer reported that Asians living in low socioeconomic status neighborhoods had a greater proportion of late-stage liver cancer [48]. These findings may be a result of higher income neighborhoods having more available resources and services that confer a health advantage.

There are several limitations to this study. We were not able to include individual-level data on English language proficiency, length of residence in the USA, or socioeconomic status that might interact with neighborhood-level factors and help explain observed results. Similarly, we have no data on health beliefs, lifestyle factors, and rates of colon cancer screening. Yet disparities in colorectal cancer screening, in particular, may contribute to observed differences in cancer stage at diagnosis, especially late-stage disease [49]. Also, we did not have data on Hispanic or Asian subgroups that might help explain results. Although these groups are often grouped together, they are not homogenous.

Despite these limitations, this paper highlights the importance of neighborhood characteristics in late-stage colorectal cancer diagnosis. Efforts are needed to reach these populations or find affordable ways to get them screened for colorectal cancer. Also, further research is needed to tease out the contribution of individual- and neighborhood-level factors on influencing uptake of colorectal cancer screening; yet collecting individual-level data through surveys can be costly and time-consuming, and cancer registry data often does not include individual-level measures of socioeconomic status, language, or acculturation. Also evidence-based interventions are needed to increase use of colorectal cancer screening and reduce late-stage diagnosis, taking into account both individual and neighborhood factors.

## 5. Conclusions

Implementation of the Affordable Care Act (ACA), which does not require copayment for colorectal cancer screening, will likely reduce the magnitude of the influence of health insurance coverage as a contributing factor to late-stage colorectal cancer diagnosis. However, only US citizens and legal residents will be able to obtain health coverage under the ACA [50]. California, home to an estimated 1.8 million undocumented immigrants aged 18–64 [51], is likely to have one of the largest populations of individuals left out of the ACA. On the other hand, studies have not consistently found that public insurance predicts a higher likelihood of early-stage diagnosis [18] but rather that complex combinations of factors influence likelihood of receiving screening in addition to insurance status and actual health care access.

## Figures and Tables

**Table 1:** Characteristics of colorectal cancer cases (ages 50+) diagnosed in California, 2005–2007.

	*N*	%	% late stage	*P* value
Age (years)				
50–75	22320	62.0	56.7	ns
75+	13710	38.0	57.0	

Race/ethnicity				
White	23612	65.5	56.0	<.0001
Hispanic/Latino	5340	14.8	58.6	
African-American	2582	7.2	60.6	
Asian	4178	11.6	58.7	
Other	318	0.9	34.0	

Gender				
Male	18239	50.6	55.6	<.0001
Female	17791	49.4	58.1	

Marital status				
Married	19892	55.2	55.5	<.0001
Not married	16138	44.8	58.5	

Health insurance				
Uninsured	545	1.5	66.8	<.0001
Private	16564	46.0	55.2	
Medicaid	1993	5.5	66.5	
Medicare	16139	44.8	56.8	
Military/veterans	236	0.7	52.5	
Unknown	553	1.5	62.8	

% limited English proficiency				
Q1 (low)	9007	25.0	56.2	.0003
Q2	9014	25.0	55.5	
Q3	8994	25.0	57.0	
Q4 (high)	9014	25.0	58.5	

% recent immigrants				
Q1 (low)	9009	25.0	55.5	<.0001
Q2	9006	25.0	56.5	
Q3	9008	25.0	56.3	
Q4 (high)	9006	25.0	59.0	

Median household income				
Q1 (low)	9004	25.0	59.4	<.0001
Q2	9010	25.0	56.8	
Q3	9006	25.0	56.2	
Q4 (high)	9010	25.0	54.8	

Neighborhood deprivation				
Q1 (low)	9006	25.0	55.2	<.0001
Q2	9004	25.0	56.7	
Q3	8997	25.0	56.3	
Q4 (high)	9010	25.0	59.0	

**Table 2:** Multivariate predictors of stage (late versus early) by race (with CI).

		All races		White		Hispanic		Black		Asian	
		OR	CI	OR	CI	OR	CI	OR	CI	OR	CI
Age	Continuous	1.01	(0.98, 1.03)	0.99	(0.97, 1.02)	1.00	(0.95, 1.06)	1.03	(0.95, 1.13)	1.05	(0.99, 1.13)

Sex	Female	1.08	(1.03, 1.13)^*∗*^	1.08	(1.03, 1.14)^*∗*^	1.05	(0.94, 1.18)	1.09	(0.92, 1.30)	1.11	(0.98, 1.27)
	Male (baseline)										

Marital status	No	1.07	(1.02, 1.12)^*∗*^	1.09	(1.03, 1.15)^*∗*^	1.02	(0.90, 1.15)	1.09	(0.91, 1.30)	1.05	(0.91, 1.21)
	Yes (baseline)										

Insurance status	Not insured	1.54	(1.28, 1.86)^*∗*^	1.42	(1.06, 1.90)^*∗*^	1.69	(1.21, 2.36)^*∗*^	1.28	(0.64, 2.56)	1.86	(1.21, 2.87)^*∗*^
	Medicaid/public	1.50	(1.36, 1.66)^*∗*^	1.47	(1.25, 1.72)^*∗*^	1.51	(1.25, 1.84)^*∗*^	1.46	(1.05, 2.02)^*∗*^	1.69	(1.35, 2.13)^*∗*^
	Medicare	1.02	(0.97, 1.08)	1.01	(0.95, 1.07)	1.10	(0.96, 1.26)	0.94	(0.78, 1.12)	1.12	(0.96, 1.31)
	Military/veterans	0.90	(0.69, 1.16)	1.06	(0.76, 1.47)	0.45	(0.22, 0.91)^*∗*^	1.13	(0.56, 2.27)	0.60	(0.22, 1.63)
	Unknown	1.41	(1.17, 1.69)^*∗*^	1.33	(1.05, 1.68)^*∗*^	1.37	(0.91, 2.06)	1.44	(0.72, 2.91)	2.02	(1.14, 3.57)^*∗*^
	Private (baseline)										

% limited English proficiency	1 low (baseline)										
	2	0.93	(0.87, 0.99)^*∗*^	0.93	(0.87, 1.00)	0.86	(0.67, 1.10)	1.17	(0.85, 1.61)	0.90	(0.67, 1.20)
	3	0.94	(0.87, 1.02)	0.93	(0.85, 1.02)	0.87	(0.65, 1.14)	1.29	(0.89, 1.87)	0.87	(0.63, 1.21)
	4 high	0.90	(0.81, 1.00)	0.95	(0.83, 1.08)	0.71	(0.51, 0.99)^*∗*^	1.31	(0.86, 1.97)	0.80	(0.55, 1.16)

% recent immigrants	1 low (baseline)										
	2	1.05	(0.98, 1.12)	1.04	(0.96, 1.12)	1.36	(1.10, 1.68)^*∗*^	0.89	(0.65, 1.21)	0.90	(0.68, 1.17)
	3	1.04	(0.96, 1.12)	1.02	(0.93, 1.12)	1.50	(1.19, 1.90)^*∗*^	0.78	(0.57, 1.09)	0.88	(0.66, 1.18)
	4 high	1.12	(1.03, 1.23)^*∗*^	1.09	(0.97, 1.22)	1.57	(1.22, 2.02)^*∗*^	0.93	(0.64, 1.33)	1.01	(0.74, 1.38)

% neighborhood deprivation	1 low (baseline)										
	2	1.02	(0.95, 1.10)	1.04	(0.96, 1.13)	1.09	(0.85, 1.39)	1.02	(0.73, 1.43)	0.88	(0.72, 1.09)
	3	0.94	(0.86, 1.03)	0.96	(0.87, 1.07)	1.00	(0.75, 1.33)	0.85	(0.59, 1.23)	0.85	(0.65, 1.12)
	4 high	0.96	(0.86, 1.07)	1.02	(0.90, 1.17)	0.95	(0.69, 1.31)	0.81	(0.55, 1.21)	0.83	(0.61, 1.14)

Median household income	1 low (baseline)										
	2	0.93	(0.87, 0.99)^*∗*^	0.98	(0.90, 1.07)	0.93	(0.80, 1.08)	0.87	(0.69, 1.09)	0.74	(0.60, 0.91)^*∗*^
	3	0.90	(0.83, 0.98)^*∗*^	0.94	(0.85, 1.04)	0.88	(0.73, 1.07)	0.88	(0.66, 1.18)	0.77	(0.62, 0.97)^*∗*^
	4 high	0.85	(0.77, 0.93)^*∗*^	0.92	(0.82, 1.03)	0.88	(0.66, 1.16)	0.73	(0.49, 1.07)	0.62	(0.47, 0.81)^*∗*^

Race/ethnicity	Asian	1.09	(1.02, 1.17)^*∗*^
	Black	1.15	(1.05, 1.25)^*∗*^
	Hispanic	1.06	(0.99, 1.14)
	White (baseline)		

^*∗*^ *P* < .05.
